# Author Correction: Discovery of fungal surface NADases predominantly present in pathogenic species

**DOI:** 10.1038/s41467-021-22476-7

**Published:** 2021-03-25

**Authors:** Øyvind Strømland, Juha P. Kallio, Annica Pschibul, Renate H. Skoge, Hulda M. Harðardóttir, Lars J. Sverkeli, Thorsten Heinekamp, Olaf Kniemeyer, Marie Migaud, Mikhail V. Makarov, Toni I. Gossmann, Axel A. Brakhage, Mathias Ziegler

**Affiliations:** 1 Department of Biomedicine, University of Bergen, Bergen, Norway; 2 Department of Molecular and Applied Microbiology, Leibniz Institute for Natural Product Research and Infection Biology, Hans Knöll Institute, Jena, Germany; 3 Department of Biological Sciences, University of Bergen, Bergen, Norway; 4 Mitchell Cancer Institute, University of South Alabama, Mobile, AL USA; 5 Department of Animal Behaviour, Bielefeld University, Bielefeld, Germany; 6 Department of Animal and Plant Sciences, University of Sheffield, Sheffield, UK; 7 Institute of Microbiology, Friedrich Schiller University Jena, Jena, Germany

**Keywords:** Hydrolases, X-ray crystallography, Fungal biology, Pathogens

Correction to: *Nature Communications* 10.1038/s41467-021-21307-z, published online 12 March 2021.

The original version of this Article omitted the following from the Acknowledgements:

We made use of the Facility for Biophysics, Structural Biology and Screening at the University of Bergen (BiSS), which has received funding from the Research Council of Norway (RCN) through the NORCRYST (grant number 245828) consortium. This work was also supported by the RCN through the Norwegian NMR Platform, NNP (226244/F50).

This has now been corrected in both the PDF and HTML versions of the Article.

