# Supplementary material for: Discovery of fungal surface NADases predominantly present in pathogenic species
Source: Nat Commun. 2021 Mar 12;12:1631. doi: 10.1038/s41467-021-21307-z (PMC7955114; doi:10.1038/s41467-021-21307-z)
Supplement: Supplementary file 1 — Supplementary Information [file 41467_2021_21307_MOESM1_ESM.pdf]

Supplementary Information

Discovery of fungal surface NADases predominantly present in pathogenic species

Øyvind Strømland[1], Juha P. Kallio[1], Annica Pschibul[2], Renate H. Skoge[3], Hulda M. Harðardóttir[3], Lars J. Sverkeli[1,3], Thorsten Heinekamp[2], Olaf Kniemeyer[2], Marie Migaud[4], Mikhail V. Makarov[4], Toni I. Gossmann[5,6], Axel Brakhage[2,7], Mathias Ziegler[1*]

Supplementary table 1. Data Collection, Phasing, and Refinement Statistics

**Structure**	**AfNADase:apo**	**AfNADase:ADPR-Nam**	**AfNADase:BAD**
PDB accession code	6YGE	6YGF	6YGG
**Data collection**			
Synchrotron/beamline	PETRAIII, EMBL/P13	PETRAIII, DESY/P11	DLS/I04-1
Space group	P 32 2 1	P 32 2 1	P 21 21 21
Cell dimensions a, b, c (Å)	63.79, 63.79, 257.99	63.82, 63.82, 257.72	51.05, 64.32, 161.01
Angles α, β, γ (°)	90, 90, 120	90, 90, 120	90, 90, 90
Resolution (Å)[a]	54.0-1.6 (1.657-1.6)	42.9-1.7 (1.76-1.7)	48.6-1.85 (1.916-1.85)
	(1.657-1.6)	(1.76-1.7)	(1.916-1.85)
Total reflections	672818 (50757)	136212 (12968)	303366 (30838)
Unique reflections	75947 (7267)	68236 (6579)	45536 (4447)
Multiplicity	8.9 (7.0)	2.0 (2.0)	6.7 (6.9)
Completeness (%)	92.8 (90.3)	99.7 (97.8)	98.5 (97.7)
I/σ(I)	16.5 (0.7)	15.2 (1.0)	9.3 (0.95)
Wilson B-factor	29.5	27.5	25.7
R-merge	5.9 (168.8)	2.5 (64.4)	14.3 (165.8)
R-pim	1.9 (61.0)	2.5 (64.4)	5.9 (67.5)
CC 1/2	0.99 (0.39)	0.99 (0.48)	0.998 (0.51)
**Refinement**			
R_{work}	17.8 (33.3)	16.9 (34.3)	18.4 (34.0)
R_{free}	21.1 (35.4)	20.1 (36.1)	22.5 (35.70)
Number of non-hydrogen atoms	3970	4173	3998
Macromolecules	3322	3346	3302
Ligands	171	263	237
Solvent	477	564	459
R.m.s.d (bonds/angles)	0.007/0.88	0.007/0.86	0.008/0.96
Ramachandran favored (%)	98.30	98.79	98.55
Ramachandran allowed (%)	1.70	1.21	1.21
Ramachandran outliers (%)	0	0	0.24
Clashscore	2.95	3.29	3.68
Average B-factor	39.8	35.2	32.7
Macromolecules	37.4	31.2	30.0
Ligands (including carbohydrates and substrates)	59.7	69.4<br>*ADPR only: 73.1*<br>*Nam only: 33.1*	54.2<br>*BAD only: 36.8*
Solvent	49.1	43.3	41.0

[a] Data for the highest resolution shell are given in parentheses

Supplementary Table 2. Structural alignment of *Af*NADase to various NADases and ADP-ribose transferase using PDBeFOLD.

Protein	PDB	Z score	RMSD (Å)	Nalgn	Seq. ID (%)
Mtb CpnT TNT	4QLP	10.7	1.93	134	27
Tse6	4ZV0	8.5	2.13	90	21
Diphtheria toxin	1TOX	5	2.71	71	11
Cholera toxin	1XTC	4.3	3.26	57	9
SPN	3PNT	3.6	3.61	73	5
PARP1	4DQY	2.9	3.48	75	9

The table shows the summary of PDBeFOLD results of pairwise structural comparison of *Af*NADase and selected NADases and ADPR transferases. The Z-score measures the significance of a match in terms of Gaussian statistics. The root-means-square deviation in angstrom (RMSD), the length of the aligned segment (Nalgn) and the fraction of identical residues in the aligned segment (Seq. ID (%)) are shown.

Supplementary Table 3. Primers used in the study.

Primer	Sequence (5'-3')
**Generation of *A. fumigatus* strains**	
6G14470_5for	GGTCATTGTAAATATCTGGG
6G14470_ptrArev	GGCCTGAGTGGCCATCGAATTCCGCCGTGTAATACTGAGAAG
6G14470_ptrAfor	GAGGCCATCTAGGCCATCAAGCCTTATGGGAAGTGGATCTTG
6G14470_3rev	GTAGTGGATAACGAAGATTCG
ptrA-for	GAATTCGATGGCCACTCAGGCC
ptrA-rev	GCTTGATGGCCTAGATGGCCTC
14470_fwd	AGGCGTATCACGAGGCCCTTTCGTCGGTCATTGTAAATATCTGGG
14470_rev	CAATAGTGCCACGCTATTGGGATCACTGGC
hph_fwd	TGATCCCAATAGCGTGGCACTATTGATCATCC
hph_rev	GGCCATCGAATTCGCCAGTGTGCTGGAATTC
compl_ptrA_fwd	CAGCACACTGGCGAATTCGATGGCCACTCAG
compl_ptrA_rev	TCACCGTCATCACCGAAACGCGCGAGCTTGATGGCCTAGATGG
**Generation of AfNADase insect expression plasmid**	
AfNADaseFw_1	CAGGGACCCGGTATGATCTTCACCAACGCCATTCTGGTGATTAGCGCC
AfNADaseRv_233	GAAACAGCACTGCCTGATTCGGCCCCGGAGTATACGGATTCG
**Generation of AfNADase 293 expression plasmid**	
AfNADase293Fw	GTTGGATCCCCACCATGATCTTCACCAAC
AfNAdase293Rv	GCATAGAATTCCTAGTGATGGTGATGGTGATGCTGATTCGGCCCCGGAGTATAC
**Generation of AfNADase mutants**	
F137AFw	GTATGGCACCGCTCTGGCGCCGC
F137ARv	TCCGATCCGAAACGGTCAAG
Q194AFw	GATGGGGACGGCTTTCGTGACATATACCAATG
Q194ARv	CCTGGCTGCTCAAACCAA
Q194KFw	GATGGGGACGAAGTTCGTGACAT
Q194KRv	CCTGGCTGCTCAAACCAAG
R129AFw	GAAGCTTGACGCGTTCGGATCGGAGTATGG
R129ARv	ATGCCAACCGGTAAGGTC
F130AFw	GCTTGACCGTGCGGGATCGGAGTATG
F130ARv	TTCATGCCAACCGGTAAG
D219A/E220AFw	GAGCGAGTATGCTGCCAAGGTGGAATACTC
D219A/E220ARv	TCATCCAACCGTCGCAAG

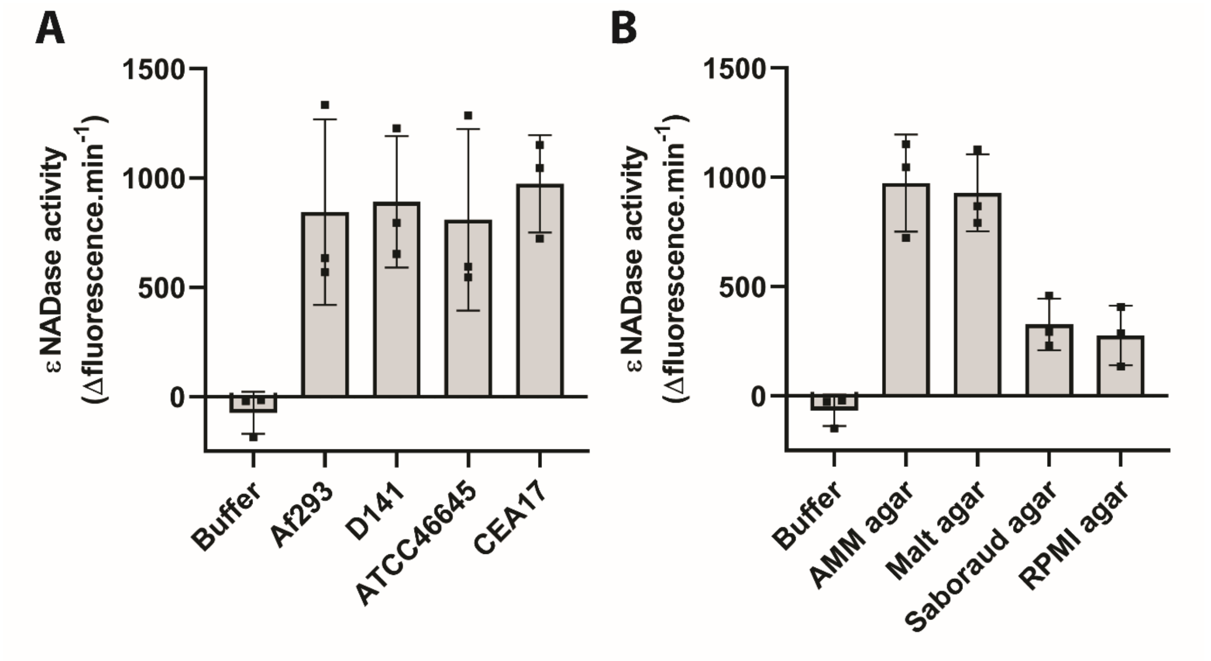

Supplementary Figure 1. NADase activity of conidia from clinical *A. fumigatus* strains and conidia grown on different media. (A) NADase activity of *A. fumigatus* conidia from the clinical isolates Af293, D141, ATCC46645 and the laboratory strain CEA17*ΔakuB* demonstrated by a fluorometric assay using εNAD, n=3. Data are presented as mean values +/- SEM. (B) NADase activity of *A. fumigatus* conidia from the strain CEA17*ΔakuB* cultivated on different media demonstrated by a fluorometric assay using εNAD, n=3. Data are presented as mean values +/- SEM. Experiments in A and B were performed independently three times with similar results. Source data are provided as a Source Data file.

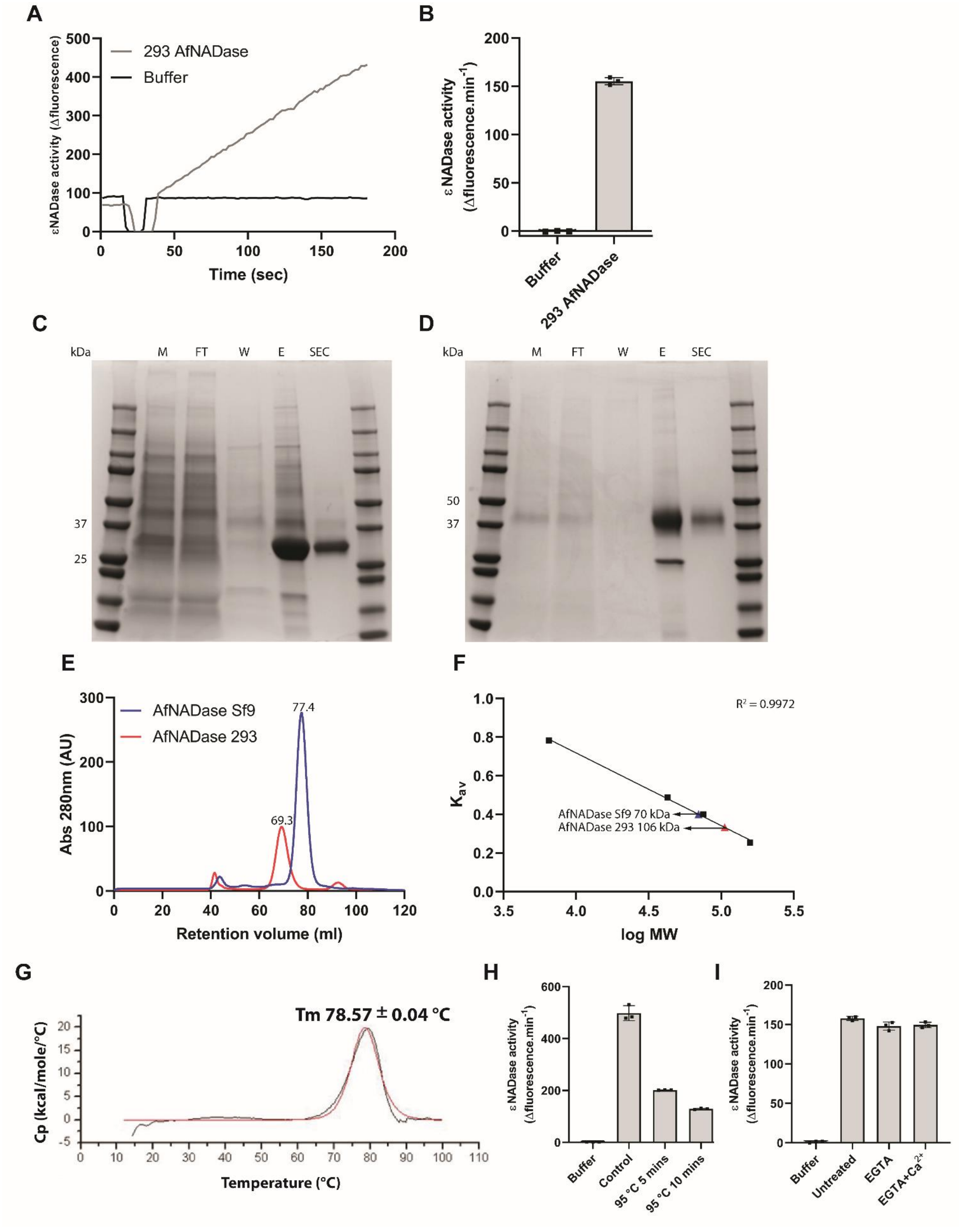

A
293 AfNADase
Buffer
εNADase activity (Δfluorescence)
Time (sec)
B
ε NADase activity (Δfluorescence.min-1)
Buffer
293 AfNADase
C
kDa
M FT W E SEC
37
25
D
kDa
M FT W E SEC
50
37
E
AfNADase Sf9
AfNADase 293
77.4
69.3
Abs 280nm (AU)
Retention volume (ml)
F
R2 = 0.9972
AfNADase Sf9 70 kDa
AfNADase 293 106 kDa
Kav
log MW
G
Tm 78.57 ± 0.04 °C
Cp (kcal/mole/°C)
Temperature (°C)
H
ε NADase activity (Δfluorescence.min-1)
Buffer
Control
95 °C 5 mins
95 °C 10 mins
I
ε NADase activity (Δfluorescence.min-1)
Buffer
Untreated
EGTA
EGTA+Ca2+

Supplementary Figure 2. **Purification and characterization of *Af*NADase expressed in 293 and Sf9 insect cells.** (A and B). NADase activity of *Af*NADase purified from 293 cells measured using εNAD, n=3. Data are presented as mean values +/- SEM. (C) Coomassie blue-stained SDS-PAGE of *Af*NADase expressed in Sf9 insect cells purified by immobilized metal affinity chromatography (IMAC) and size exclusion chromatography (SEC). M: medium, FT: flow through, W: wash, E: Elution from IMAC column, SEC: pooled fraction from the SEC column n=3. (D) Coomassie blue-stained SDS-PAGE showing *Af*NADase expressed in 293 cells purified by IMAC and size exclusion chromatography. M: medium, FT: flow through, W: wash, E: Elution from IMAC column, SEC: pooled fraction from the SEC column n=3. (E) Size exclusion chromatography elution profiles of *Af*NADase purified from 293 and Sf9 cells. (F) The size of *Af*NADase purified from 293 and Sf9 cells was determined by comparing their partition coefficient (K_{av}) to a standard of proteins with known sizes (E and F). NADase activity of *Af*NADase purified from 293 cells measured using εNAD, n=3. Data are presented as mean values +/- SEM. (G) Differential scanning calorimetry of *Af*NADase purified from Sf9 cells, black: experimental data, red: fitted model. Experiments in A-G were performed independently three times with similar results. (H) NADase activity of *Af*NADase following incubation at 95 °C for 5 and 10 minutes, n=3. Data are presented as mean values +/- SEM. (I) NADase activity of recombinant *Nc*NADase from S293 cells treated with EGTA or EGTA and calcium chloride, n=3. Data are presented as mean values +/- SEM. Source data are provided as a Source Data file.

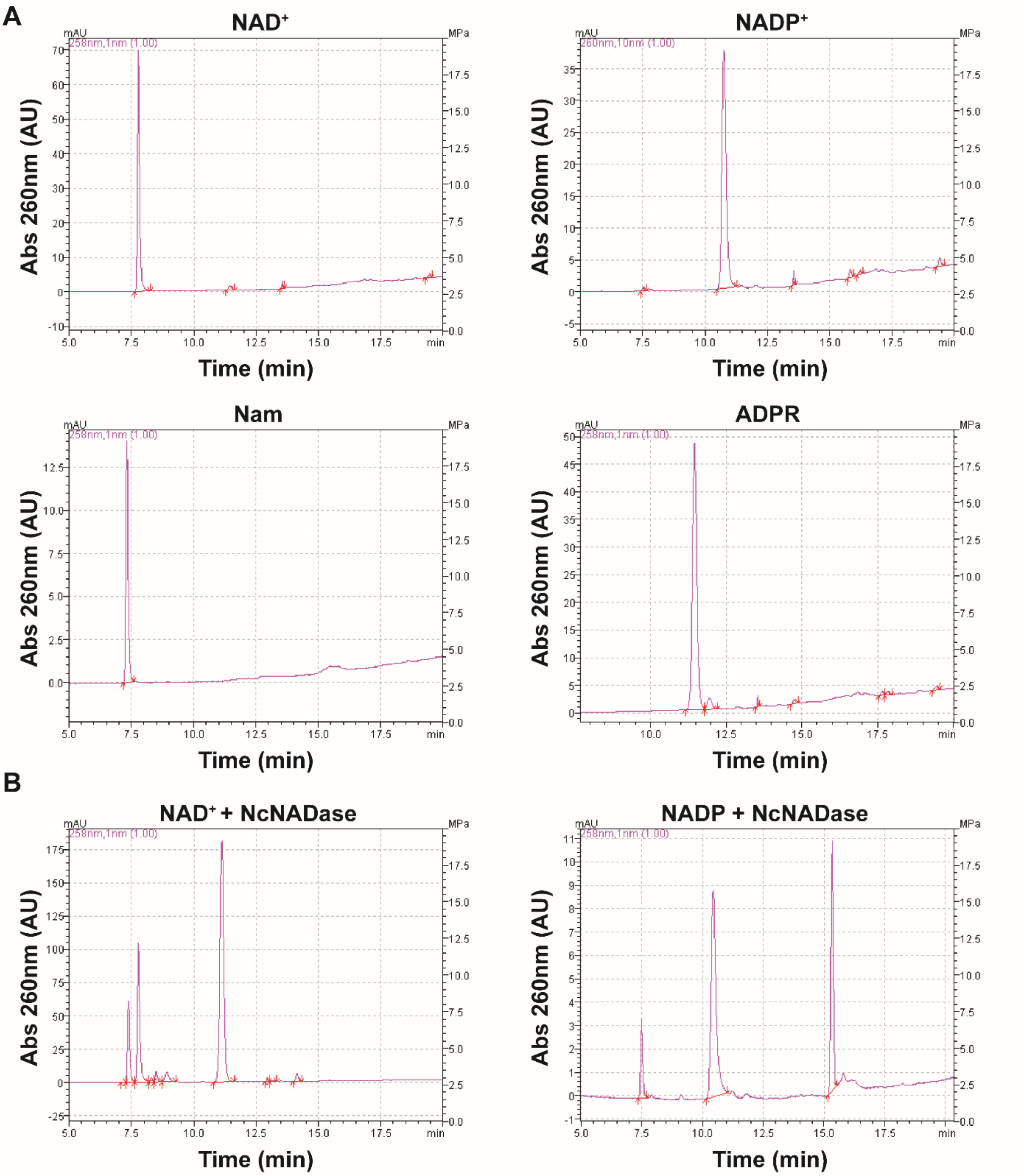

Supplementary Figure 3. HPLC elution profile of NAD$^+$, NADP$^+$ and *Nc*NADase hydrolysis products. (A) NAD$^+$, NADP$^+$, Nam and ADPR standards (B) NAD$^+$ and NADP$^+$ hydrolysis mediated by recombinant *Nc*NADase expressed and purified from 293 cells. The experiment was repeated independently three times with similar results.

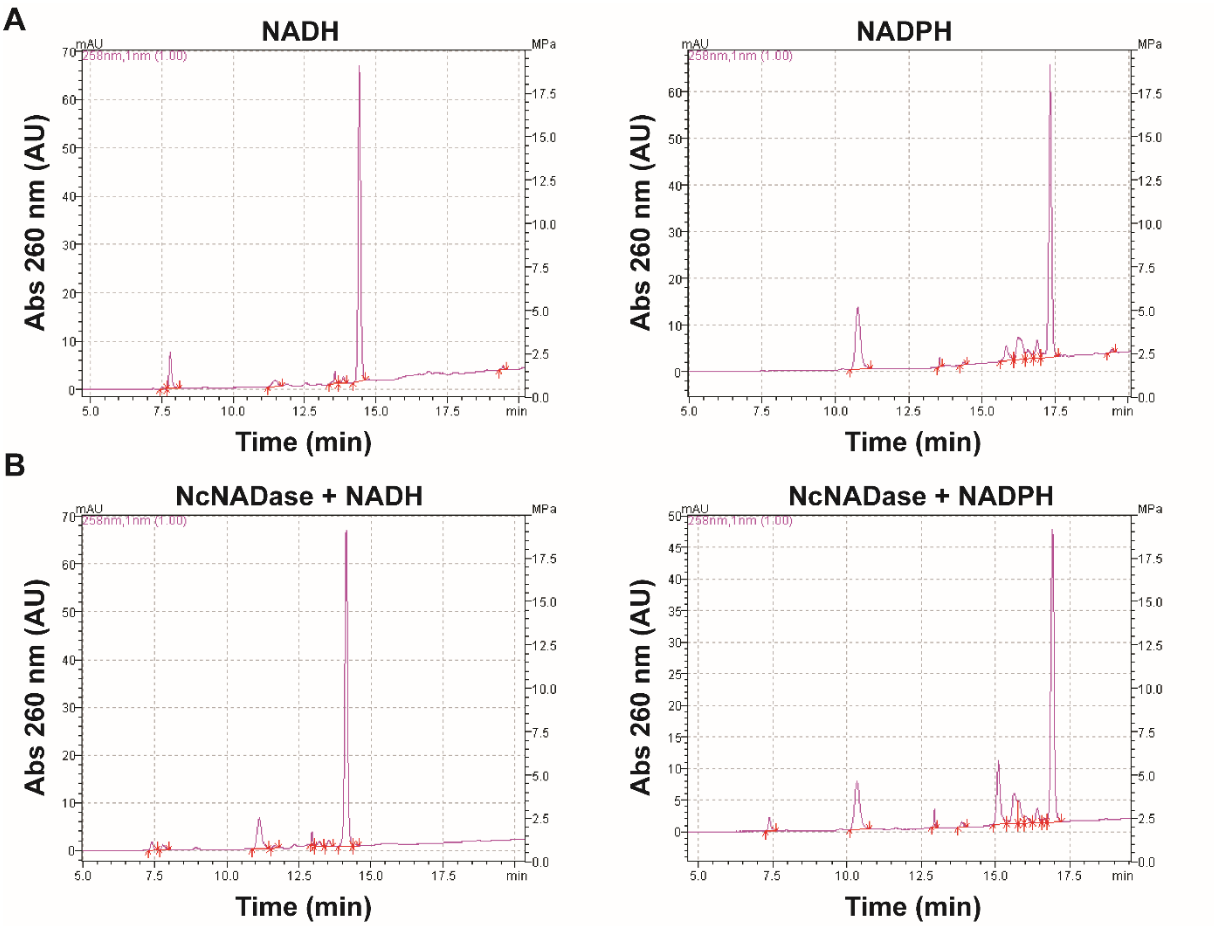

Supplementary Figure 4. HPLC elution profiles of NADH, NADPH and *Nc*NADase hydrolysis products. (A) NADH and NADPH standards (B) NADH and NADPH incubated with *Nc*NADase expressed and purified from 293 cells. The experiment was repeated independently three times with similar results.

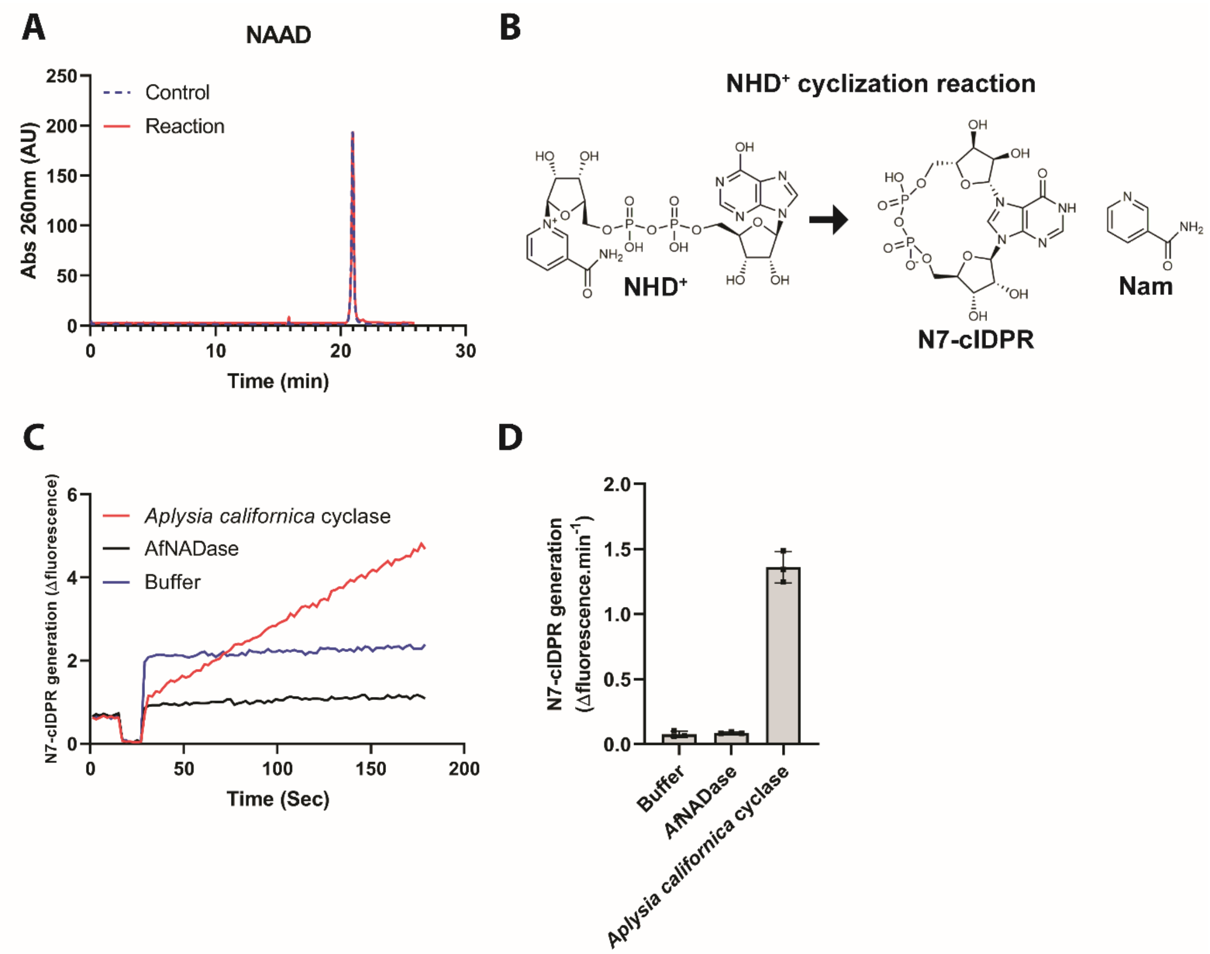

Supplementary Figure 5. *Af*NADase does not cleave NAAD or catalyse ADPR cyclization. (A) No *Af*NADase mediated reaction is observed with NAAD as shown by HPLC analysis. (B) Cyclization of NHD^+ leads to the formation of Nam and the fluorescent molecule N7-cIDRP. (C) N7-cIDPR is produced by *Aplysia californica* cyclase but not by *Af*NADase, as evidenced by fluorescence measurements, n=3. (D) Rate of N7-cIDPR formation by *Af*NADase and *A. californica* cyclase. Data are presented as mean values +/- SEM. The *A. californica* cyclase was used as a positive control. Experiments in A, C and D were performed independently three times with similar results. Source data are provided as a Source Data file.

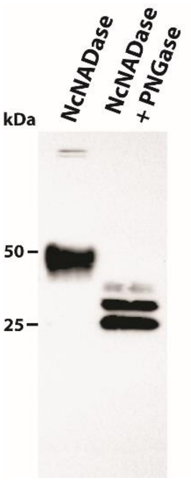

Supplementary Figure 6. Recombinant *N. crassa* NADase is N-glycosylated. Deglycosylation of purified FLAG-tagged *Nc*NADase expressed in 293 cells with Peptide-N-Glycosidase F (PNGase) reduces the molecular mass of the protein, shown by western blotting using Anti-FLAG M2 antibody. The experiment was repeated independently three times with similar results. Source data are provided as a Source Data file.

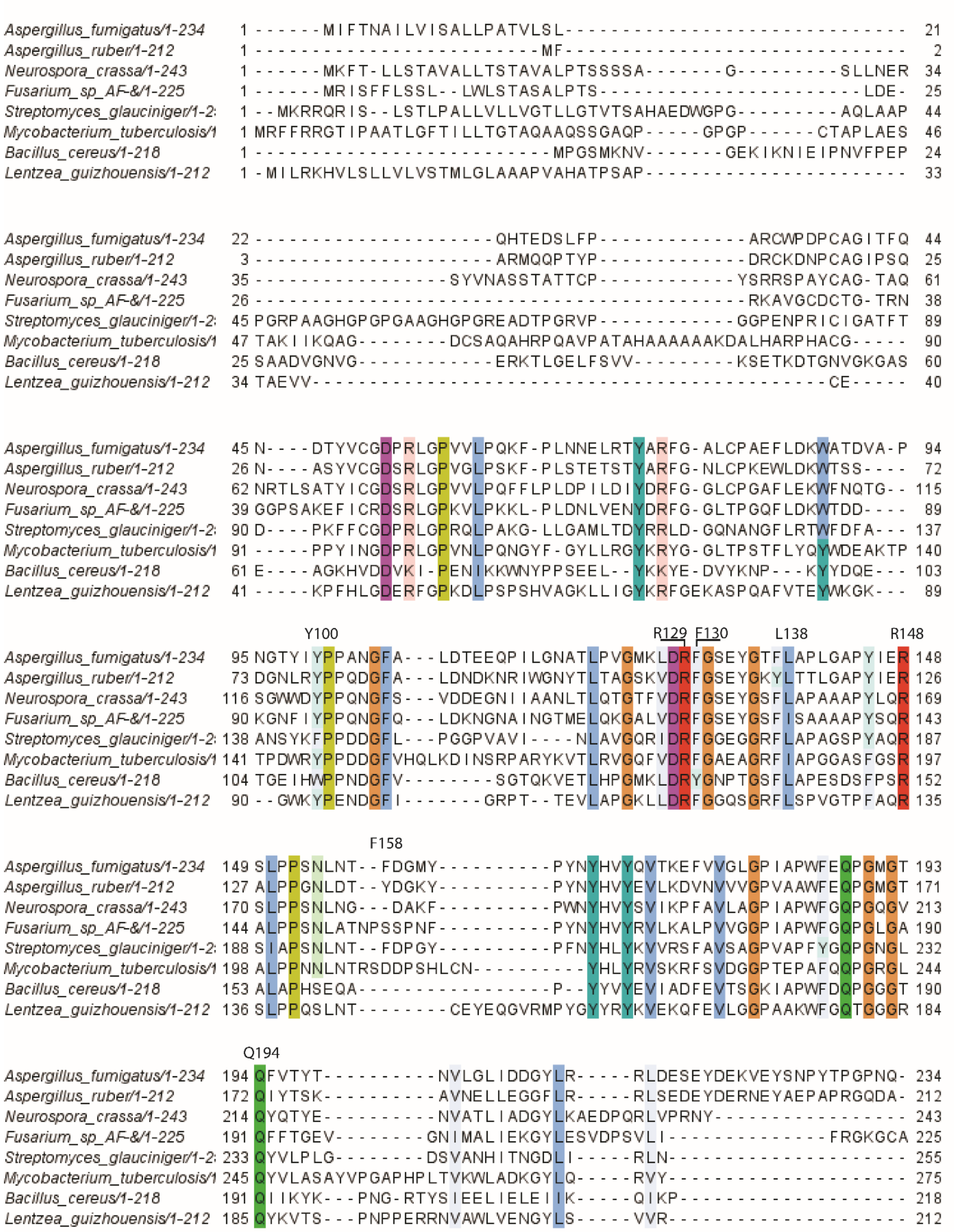

Supplementary Figure 7. Multiple sequence alignment of fungal and bacterial TNT domain-containing proteins. Multiple sequence alignment of selected TNT-containing proteins using MAFFT. Residues involved in BAD and/or product interaction in *Af*NADase are highlighted.

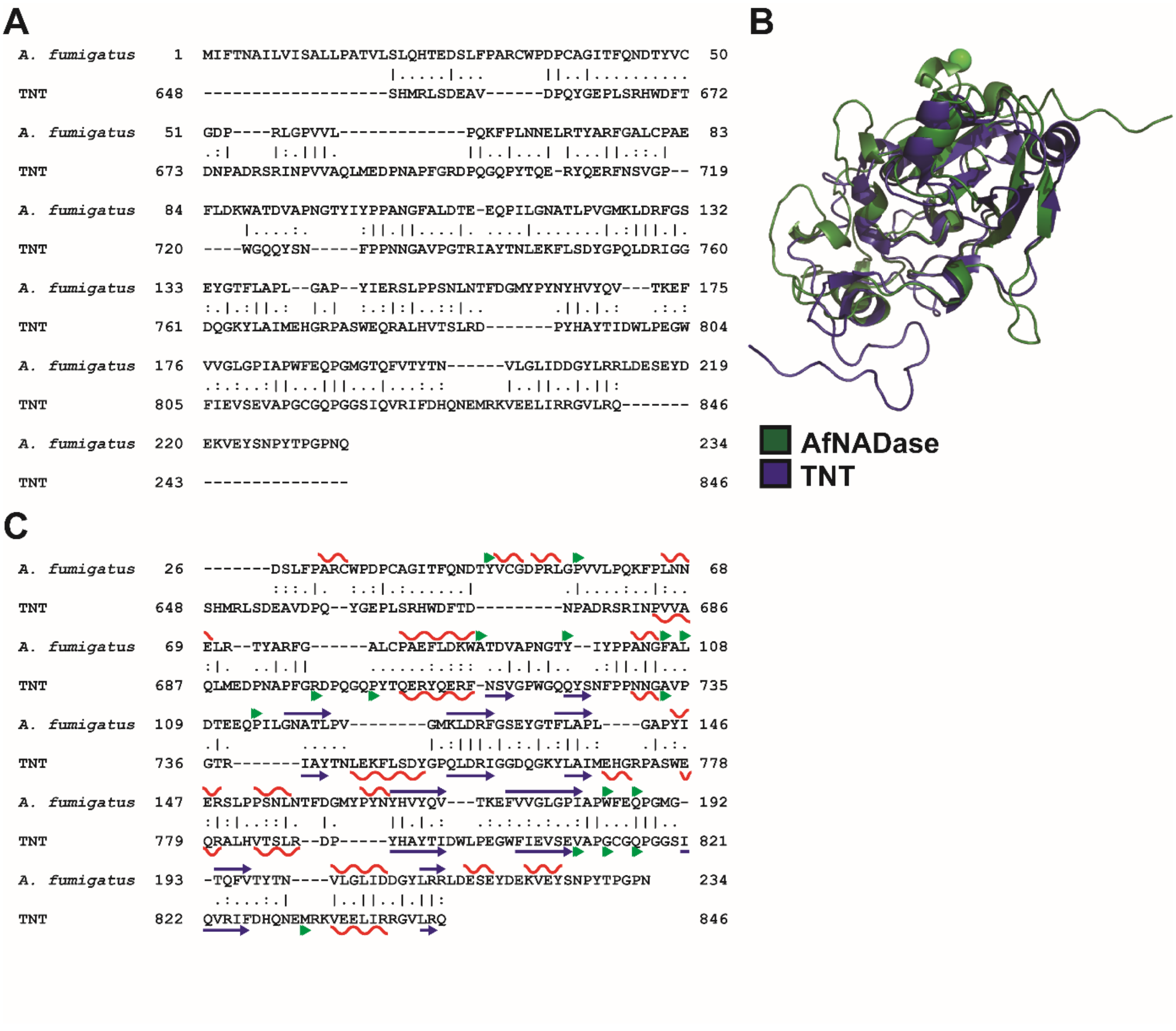

Supplementary Figure 8. Pairwise and structural alignment of *Af*NADase and TNT. (A) Global pairwise alignment of *Af*NADase and the TNT domain of *Mtb* CpnT using EMBOSS Needle. (B) Structural alignment of *Af*NADase and the TNT domain of *Mtb* CpnT. (C) Structure-based sequence alignment of *Af*NADase and the TNT domain of *Mtb* CpnT. Secondary structure elements were assigned using the DSSP algorithm, the red waves indicate helical secondary structure while the blue arrows and green arrowheads indicate beta strands and beta bridges, respectively.

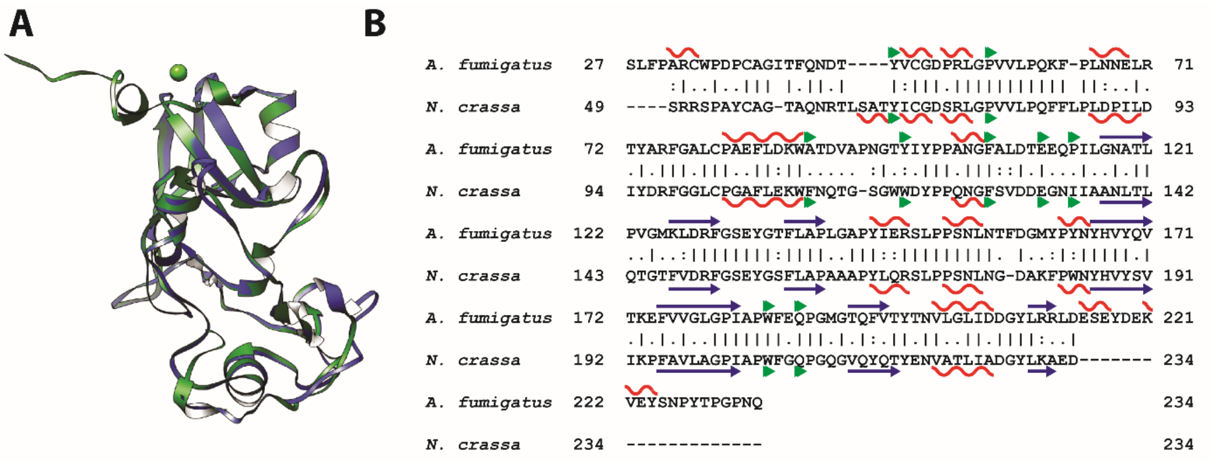

Supplementary Figure 9. Structural alignment of *Af*NADase and *Nc*NADase (A) Structural alignment of *Af*NADase (green) and the model of *Nc*NADase (blue). (B) Structure-based sequence alignment of *Af*NADase and the model of *Nc*NADase. Secondary structure elements were assigned using the DSSP algorithm; the red waves indicate helical secondary structure while the blue arrows and green arrowheads indicate beta strands and beta bridges, respectively.
