# Supplementary material for: Discovery of fungal surface NADases predominantly present in pathogenic species
Source: Nat Commun. 2021 Mar 12;12:1631. doi: 10.1038/s41467-021-21307-z (PMC7955114; doi:10.1038/s41467-021-21307-z)
Supplement: Supplementary file 3 — Description of Additional Supplementary Files [file 41467_2021_21307_MOESM3_ESM.pdf]

Description of Additional Supplementary Files

File Name: Supplementary Data 1
Description: Proteins identified by mass spectrometry in fluorescent bands excised from the activity gels in Figure 1D.
