# Supplementary material for: Discovery of fungal surface NADases predominantly present in pathogenic species
Source: Nat Commun. 2021 Mar 12;12:1631. doi: 10.1038/s41467-021-21307-z (PMC7955114; doi:10.1038/s41467-021-21307-z)
Supplement: Supplementary file 5 — Reporting Summary [file 41467_2021_21307_MOESM5_ESM.pdf]

nature research

Corresponding author(s): Mathias Ziegler

Last updated by author(s): Dec 11, 2020

Reporting Summary

Nature Research wishes to improve the reproducibility of the work that we publish. This form provides structure for consistency and transparency in reporting. For further information on Nature Research policies, see Authors & Referees and the Editorial Policy Checklist .

Statistics

For all statistical analyses, confirm that the following items are present in the figure legend, table legend, main text, or Methods section.

n/a	Confirmed	
☐	☒	The exact sample size (n) for each experimental group/condition, given as a discrete number and unit of measurement
☒	☐	A statement on whether measurements were taken from distinct samples or whether the same sample was measured repeatedly
☒	☐	The statistical test(s) used AND whether they are one- or two-sided<br>*Only common tests should be described solely by name; describe more complex techniques in the Methods section.*
☒	☐	A description of all covariates tested
☒	☐	A description of any assumptions or corrections, such as tests of normality and adjustment for multiple comparisons
☐	☒	A full description of the statistical parameters including central tendency (e.g. means) or other basic estimates (e.g. regression coefficient) AND variation (e.g. standard deviation) or associated estimates of uncertainty (e.g. confidence intervals)
☒	☐	For null hypothesis testing, the test statistic (e.g. F, t, r) with confidence intervals, effect sizes, degrees of freedom and P value noted<br>*Give P values as exact values whenever suitable.*
☒	☐	For Bayesian analysis, information on the choice of priors and Markov chain Monte Carlo settings
☒	☐	For hierarchical and complex designs, identification of the appropriate level for tests and full reporting of outcomes
☒	☐	Estimates of effect sizes (e.g. Cohen's d, Pearson's r), indicating how they were calculated

Our web collection on statistics for biologists contains articles on many of the points above.

Software and code

Policy information about availability of computer code

Data collection	MxCUBE was used for crystallographic data collection at P13 (DESY,EMBL) and I04-1 (Dimond light source). Custom-designed graphical user interface (P11 GUI) was used for crystallographic data collection at P11 (DESY). TopSpin (Bruker v 3.5.7) was used for collection of NMR data. MS data was aquired using the Xcalibur™ software suite (Thermo Scientific v 4.2).
Data analysis	Primers for mutagenesis was generated using NEBaseChanger (v. 1.3.0). The phylogenetic tree was generated using TimeTree (http://timetree.org/, accessed: 05.12.2019) Crystallographic data analysis was performed with XDS (versions 2017-2019), CCP4 (v 7, AIMLESS), PHENIX (v 1.17, including REFINE, MolProbity and eLBOW), Coot (v 0.8), PyMOL (v 1.8), UCSF Chimera (v 1.14) NMR data were analysed using TopSpin (v 3.5.7) and Dynamics Center (v 2.5), the NMR data was fitted using MatLab (v 2019a) . MS data was analysed using Proteome Discoverer 2.2 and the algorithms of Mascot 2.4.1, Sequest HT, and MS Amanda 2.0. Adobe Illustrator (v CS5), Photoshop (v CS5) and GraphPad (v 8.1.2) were used for final figure preparation.

For manuscripts utilizing custom algorithms or software that are central to the research but not yet described in published literature, software must be made available to editors/reviewers. We strongly encourage code deposition in a community repository (e.g. GitHub). See the Nature Research guidelines for submitting code & software for further information.

Data

Policy information about availability of data

All manuscripts must include a data availability statement. This statement should provide the following information, where applicable:

- Accession codes, unique identifiers, or web links for publicly available datasets
- A list of figures that have associated raw data
- A description of any restrictions on data availability

The sequence data for Aspergillus fumigatus and Neurospora crassa were deposited in the NCBI GenBank with the accession codes MT276230 and MT316195, respectively. Crystallography atomic coordinates and structure factors were deposited in the Protein Data Bank (PDB) with accession codes 6YGE, 6YGF and 6YGG. All other data are included in the paper and any further information will be provided upon request.

Field-specific reporting

Please select the one below that is the best fit for your research. If you are not sure, read the appropriate sections before making your selection.

☒ Life sciences ☐ Behavioural & social sciences ☐ Ecological, evolutionary & environmental sciences

For a reference copy of the document with all sections, see nature.com/documents/nr-reporting-summary-flat.pdf

Life sciences study design

All studies must disclose on these points even when the disclosure is negative.

Sample size	No statistical method was applied to predetermine the sample size. No inference from small sample to larger populations. The sample size was selected based on the standard in the field. Experiments presented in this study were repeated to confirm reproducibility.
Data exclusions	No data were excluded.
Replication	Biochemical experiments were repeated three times independently with similar results, all attemps of replication were successfull. Several datasets were collected from different crystals but only the datasets with the highest resolution were solved and refined.
Randomization	Samples were not allocated into experimental groups, so randomization is not applicable.
Blinding	Blinding was not relevant to the study as the results could not be influenced by subjective bias. Bias introduced during crystallographic model building is accounted for by the statistical analysis incorporated into the refinement procedure. This data is included in Supplementary Table S1.

Reporting for specific materials, systems and methods

We require information from authors about some types of materials, experimental systems and methods used in many studies. Here, indicate whether each material, system or method listed is relevant to your study. If you are not sure if a list item applies to your research, read the appropriate section before selecting a response.

Materials & experimental systems

n/a	Involved in the study	
☐	☒	Antibodies
☐	☒	Eukaryotic cell lines
☒	☐	Palaeontology
☒	☐	Animals and other organisms
☒	☐	Human research participants
☒	☐	Clinical data

Methods

n/a	Involved in the study	
☒	☐	ChIP-seq
☒	☐	Flow cytometry
☒	☐	MRI-based neuroimaging

Antibodies

Antibodies used	Monoclonal ANTI-FLAG® M2 antibody produced in mouse (F3165, Sigma-Aldrich, Lot: SLBL123V7)
Validation	The manufacturer validated the purity of the antibody by SDS-PAGE. The manufacturer validated the sensitivity by detection of 2 ng FLAG-BAP fusion protein using dot blot. The manufacturer validated the specificity by detection of a single band of protein on a western blot from an E. coli crude cell lysate.

Eukaryotic cell lines

Policy information about cell lines

Cell line source(s)	293 cells were obtained from ATCC (CRL-1573). Sf9 cells were obtained from Thermo Fischer (B82501), Neurospora Crassa conidia (strain: 2489) were a gift from professor Peter Ruoff at the University of Stavanger, Norway. Aspergillus fumigatus (strain: CEA17ΔakuB) was generated earlier, see method section.
Authentication	The 293 Cell line was authenticated with morphology, karyotyping, and PCR based approaches by ATCC. The other cell lines were not validated
Mycoplasma contamination	The 293 cell line was tested for mycoplasma contamination and confirmed as mycoplasma negative. The other cell lines were not tested.

Commonly misidentified lines<br>(See ICLAC register)	No commonly misidentified cell lines were used.
